# DAILY EMOTIONAL AND PHYSICAL WELL-BEING PREDICTED BY PERSONAL AND OTHER VIEWS OF AGING

**DOI:** 10.1093/geroni/igac059.901

**Published:** 2022-12-20

**Authors:** Shevaun Neupert, Reyyan Can

**Affiliations:** North Carolina State University, Raleigh, North Carolina, United States; North Carolina State University, Raleigh, North Carolina, United States

## Abstract

Individual differences in personal views of aging and how older adults perceive others contribute to health. We examined these relationships as they unfold within persons over time. A 14-day daily diary study of 428 participants aged 50-85 (M = 63.51) assessed subjective age (how old one feels), ageism, negative affect, and physical health each day. Increases in daily subjective age corresponded to increases in negative affect, but there was no effect of daily ageism. In contrast, increases in daily subjective age and daily ageism were each uniquely associated with increases in daily physical health problems. Further, these views of aging interacted; daily increases in ageism were especially detrimental to physical health when accompanied by increases in subjective age. These results suggest that daily fluctuations in personal views of aging are important for emotional and physical well-being, whereas personal views as well as perceptions of others work together for physical well-being.

